# The Regulator PltZ Regulates a Putative ABC Transporter System PltIJKNOP of *Pseudomonas aeruginosa* ATCC 27853 in Response to the Antimicrobial 2,4-Diacetylphloroglucinol

**DOI:** 10.3389/fmicb.2020.01423

**Published:** 2020-07-08

**Authors:** Ding-Ding Guo, Li-Ming Luo, Hai-Long Ma, Si-Ping Zhang, Hang Xu, Honghua Zhang, Yong Wang, Yongna Yuan, Zhen Wang, Yong-Xing He

**Affiliations:** ^1^School of Pharmacy, Lanzhou University, Lanzhou, China; ^2^Ministry of Education, Key Laboratory of Cell Activities and Stress Adaptations, School of Life Sciences, Lanzhou University, Lanzhou, China; ^3^School of Information Science and Engineering, Lanzhou University, Lanzhou, China

**Keywords:** antibiotic resistance, *P. aeruginosa*, pathogens, multidrug resistance, pyoluteorin, 2, 4-diacetylphloroglucinol, *P. fluorescens*, TetR regulator

## Abstract

*Pseudomonas aeruginosa* is an opportunistic pathogen commonly infecting immunocompromised patients with diseases like cystic fibrosis (CF) and cancers and has high rates of recurrence and mortality. The treatment efficacy can be significantly worsened by the multidrug resistance (MDR) of *P. aeruginosa*, and there is increasing evidence showing that it is easy for this pathogen to develop MDR. Here, we identified a gene cluster, *pltZ-pltIJKNOP*, which was originally assumed to be involved in the biosynthesis of an antimicrobial pyoluteorin, significantly contributing to the antibiotic resistance of *P. aeruginosa* ATCC 27853. Moreover, the TetR family regulator PltZ binds to a semi-palindromic sequence in the promoter region of the *pltIJKNOP* operon and recognizes the antimicrobial 2,4-diacetylphloroglucinol (2,4-DAPG), which in turn induces the expression of the *pltIJKNOP* operon. Using quantitative proteomics method, it was indicated that the regulator PltZ also plays an important role in maintaining metabolic hemostasis by regulating the transporting systems of amino acids, glucose, metal ions, and bacteriocins.

## Introduction

*Pseudomonas aeruginosa* is an opportunistic pathogen that infects a wide range of hosts, including plants and animals (Rahme et al., 1995). As a plant pathogen, it was reported to infect lettuce (Paine and Branfoot, 1924; Elrod and Braun, 1942), sugarcane (Desai, 1935), and tobacco (Yu et al., 2008). Most prominently, infection of *P. aeruginosa* is common in immunocompromised patients with diseases like cystic fibrosis (CF) and cancers and has high rates of recurrence and mortality (Emerson et al., 2002). The treatment efficacy can be significantly worsened by the multidrug resistance (MDR) of *P. aeruginosa*, and there is increasing evidence showing that it is easy for this pathogen to develop MDR by acquiring transferable resistance genes *via* horizontal gene transfer (Hirsch and Tam, 2010; Botelho et al., 2019). Moreover, the genomes of *P. aeruginosa* encode several resistance–nodulation–cell division (RND)-type efflux systems, four of which, i.e., MexAB-OprM, MexCD-OprJ, MexEF-OprN, and MexXY-OprM, are characterized to be essential for MDR (Poole, 2011). Many clinical MDR isolates of *P. aeruginosa* have been shown to have upregulated expressions of these efflux systems, but it is unknown whether there are still yet-unidentified efflux pumps responsible for the MDR phenotypes of *P. aeruginosa* strains. Understanding the MDR mechanism is of primary priority for the development of new therapies for treating *P. aeruginosa* infection.

Interactions of *P. aeruginosa* with other co-colonizing microbes have been proven to be critical in driving its adaptation and evolutionary changes (McGuigan and Callaghan, 2015; Tognon et al., 2017; Flynn et al., 2019). In CF lungs, where *P. aeruginosa* and other pathogenic microbes such as *Staphylococcus aureus* and *Haemophilus influenzae* coexist, the interspecies interactions have been widely investigated, and it was revealed that the quorum sensing signals played an essential role in mediating the interspecies communication and formation of multispecies biofilm, which promoted survival and enhanced virulence (Mengeloglu et al., 2013; Cohen et al., 2016). Although the plant rhizosphere has been suggested to serve as the natural habitat and dissemination agent of *P. aeruginosa* strains, it is still largely unknown how *P. aeruginosa* interacts with other root-colonizing microorganisms and how these interactions shaped the important phenotypic traits, such as antibiotic resistance, in *P. aeruginosa*.

Certain strains of *P. aeruginosa* (M18, PACS88, and PACS171b) produce a broad-spectrum antibiotic named pyoluteorin (Huang et al., 2004; Kidarsa et al., 2011), which shows toxicity against bacteria and fungi, potentially providing an advantage in competitive colonization. The gene cluster related to pyoluteorin biosynthesis comprises two pairs of oppositely transcribed operons, *pltRM-pltLABCDEFG* and *pltZ-pltIJKNOP*. The *pltLABCDEFG* operon is responsible for pyoluteorin biosynthesis and is activated by the LysR-type regulator PltR (Li et al., 2012). The *pltIJKNOP* operon, which encodes an ATP-binding cassette (ABC) transporter, was assumed to be involved in pyoluteorin efflux (Brodhagen et al., 2005). Interestingly, pyoluteorin can also be produced by some strains of *Pseudomonas protegens* (Pf-5, CHA0, and H78) (Paulsen et al., 2005; Jousset et al., 2014; Huang et al., 2017), which are root-colonizing bacteria well known for the capability of producing a wide array of antimicrobials. In these bacterial strains, an antimicrobial, 2,4-diacetylphloroglucinol (2,4-DAPG), functions as a signaling molecule to repress the production of pyoluteorin through an unknown mechanism (Baehler et al., 2005). The gene cluster responsible for 2,4-DAPG biosynthesis also consists of two pairs of oppositely transcribed operons, *phlF*-*phlACBDE* and *phlG*-*phlH*. The 2,4-DAPG biosynthetic operon *phlACBDE* is transcriptionally repressed by the TetR-type regulator PhlF, which is released from DNA upon binding 2,4-DAPG (Delany et al., 2000). The *phlG* gene encodes a 2,4-DAPG hydrolase, whose expression is regulated by the other TetR-type regulator, PhlH, in response to 2,4-DAPG (Yan et al., 2017). Notably, PhlH shares moderate sequence identity (∼39%) with the PltZ regulator encoded in the pyoluteorin-related gene cluster *pltZ-pltIJKNOP*; however, the biological function and the mechanism of action are still elusive. In this work, we found that the gene cluster *pltZ-pltIJKNOP* is involved in the resistance of *P. aeruginosa* ATCC 27853 to chloramphenicol and ampicillin. Moreover, the TetR family regulator PltZ was shown to bind a semi-palindromic sequence in the promoter region of the *pltIJKNOP* operon and recognize the antimicrobial 2,4-DAPG, which in turn induces the expression of the *pltIJKNOP* operon.

## Results

### *Pseudomonas aeruginosa* ATCC 27853 Is Defective in Pyoluteorin Biosynthesis

**FIGURE 1:**
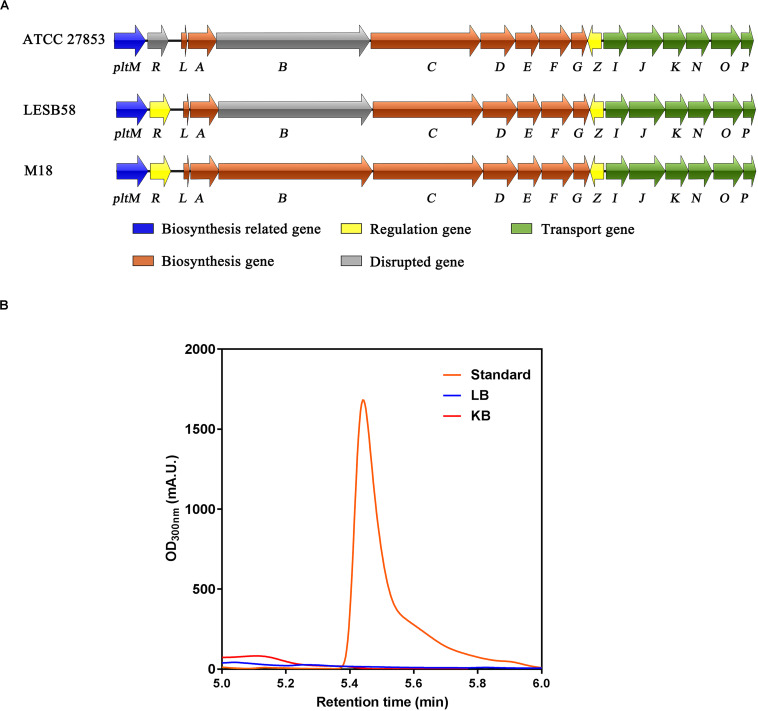
Biosynthetic gene cluster and production of pyoluteorin in *Pseudomonas aeruginosa* ATCC 27853. **(A)** Pyoluteorin biosynthetic gene cluster in *P. aeruginosa* ATCC 27853, *P. aeruginosa* LESB58, and *P. aeruginosa* M18. *Arrows* denote gene location and orientation and are colored according to molecular functions, except for the disrupted genes (in *gray*) caused by premature stop codons. **(B)** HPLC chromatograms of the supernatants of Luria–Bertani (*blue*) and King’s B (*red*) cultures inoculated with *P. aeruginosa* ATCC 27853 and the purchased pyoluteorin standard (*orange*).

Although *P. aeruginosa* M18, PACS171b, and PACS88A were shown to produce pyoluteorin, genomic analysis of *P. aeruginosa* LESB58, a highly virulent clinical strain isolated from CF patients, revealed that the *pltB* gene within the *plt* biosynthetic gene cluster *pltRM-pltLABCDEFG* had a 5-bp deletion resulting in a truncated PltB protein and a defect in pyoluteorin biosynthesis (Winstanley et al., 2009). The genomes of *P. aeruginosa* PAO1 and PA14 were also analyzed, but no *plt* gene clusters were found in these two strains. We next conducted a sequence analysis of the *plt* gene cluster in *P. aeruginosa* ATCC 27853, which was isolated from a blood specimen, and found that both *pltR* and *pltB* have premature stop codons (Figure 1A), suggesting that *P. aeruginosa* ATCC 27853 also lost the ability of producing pyoluteorin throughout evolution. By using high-performance liquid chromatography (HPLC), we confirmed that *P. aeruginosa* ATCC 27853 did not produce pyoluteorin under any of the conditions tested (Figure 1B). This suggested that the *pltZ-pltIJKNOP* operon was unlikely to be involved in transporting the endogenous pyoluteorin in *P. aeruginosa* ATCC 27853, and the biological function of this operon remains to be clarified.

### The *pltZ* Gene Negatively Regulates the *pltIJKNOP* Operon Encoding an ABC Transporter

**FIGURE 2:**
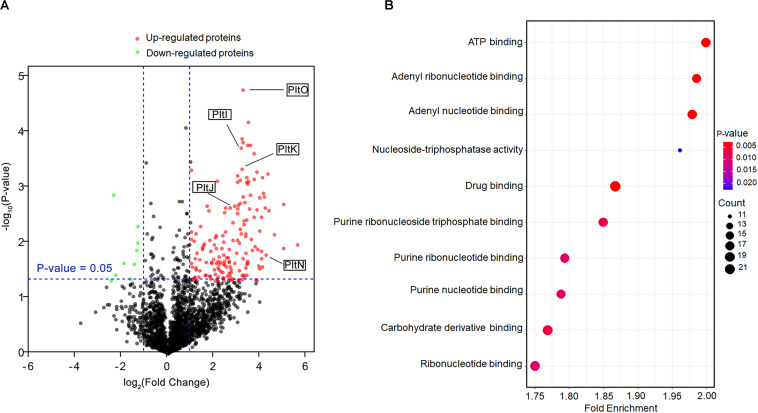
Comparative proteomic analysis of the wild-type and Δ*pltZ* strains. **(A)** Volcano plot representing the protein expression ratios of the wild-type vs. the Δ*pltZ* strain. For each protein, the –log_10_ (*p*-value) is plotted against its log_2_ (fold change). Proteins that were upregulated (*p* < 0.05, fold change > 2) in the Δ*pltZ* strain are in *red* while those downregulated (*p* < 0.05, fold change ≤ 2) are in *green*. **(B)** Top 10 hallmark pathways highlighting the differentially expressed protein pathways between the Δ*pltZ* and wild-type strains. The *x*-axis is the enrichment factor, the *spot size* denotes the protein number, and the *spot color* denotes the *p*-value.

To probe the function of the TetR family regulator PltZ, we constructed the Δ*pltZ* mutant and performed label-free quantitative proteomics to investigate the differentially expressed proteins between the Δ*pltZ* strain and the wild-type strain. In total, 2,478 proteins were identified. Student’s *t*-tests were performed with a threshold *p* < 0.05, resulting in 248 proteins quantified with high confidence. Using the criteria of a Δ*pltZ*/wild-type fold change <−2 or >2, we identified seven downregulated and 165 upregulated proteins in the Δ*pltZ* strain (Figure 2A). A Gene Ontology (GO) enrichment analysis revealed that these differentially expressed proteins were mainly involved in ATP binding, nucleotide binding, drug binding, and carbohydrate derivative binding (Figure 2B). Prominently, the expressions of PltI, PltJ, PltK, PltN, and PltO are significantly upregulated in the Δ*pltZ* strain, suggesting that PltZ acts as a repressor of the *pltIJKNOP* operon, whose function was previously suggested to be related with the efflux of pyoluteorin. The protein products of the biosynthetic *pltLABCDEFG* were not identified in the proteomic data, consistent with our finding that *P. aeruginosa* ATCC 27853 did not produce pyoluteorin (Huang et al., 2004). Sequence analysis indicated that the PltI, PltJ, and PltP proteins were similar to the three components of the type I secretion system, which comprised an ABC transporter, a membrane fusion protein (MFP), and an outer membrane protein (OMP). Structural prediction using the Phyre2 server (Kelley et al., 2015) further revealed that PltK is structurally similar to the transmembrane domain of the ABC transporter and that PltN together with PltO are structurally similar to the electron transporter CcdA belonging to the LysE superfamily (Zhou and Bushweller, 2018). However, it is currently unknown how the six translation products of the *pltIJKNOP* operon are assembled across the double membranes of *P. aeruginosa* ATCC 27853. Additionally, proteins involved in putrescine metabolism and transport were also upregulated in the Δ*pltZ* strain (Supplementary Table S1). Both Ga0133450_116399 and Ga0133450_113821 encode a glutamate–putrescine ligase, which is the first enzyme in the primary putrescine utilization pathway, while spuH (Ga0133450_11320) forms the transmembrane channel involved in putrescine uptake. Moreover, the upregulated proteins include a large number of components of transporting systems involved in the uptake of potassium (Ga0133450_114483), amino acids (Ga0133450_115924 and Ga0133450_113735), and glucose (Ga0133450_111819 and Ga0133450_111821) and the efflux of microcin (Ga0133450_113522) and cadmium (Ga0133450_113780), implying an essential role of PltZ in maintaining metabolic homeostasis. These findings indicated that the TetR family regulator PltZ exerts global influences over the physiological processes of *P. aeruginosa* ATCC 27853.

### PltZ Represses the Expression of the *pltIJKNOP* Operon by Binding a Semi-Palindromic Sequence

**FIGURE 3:**
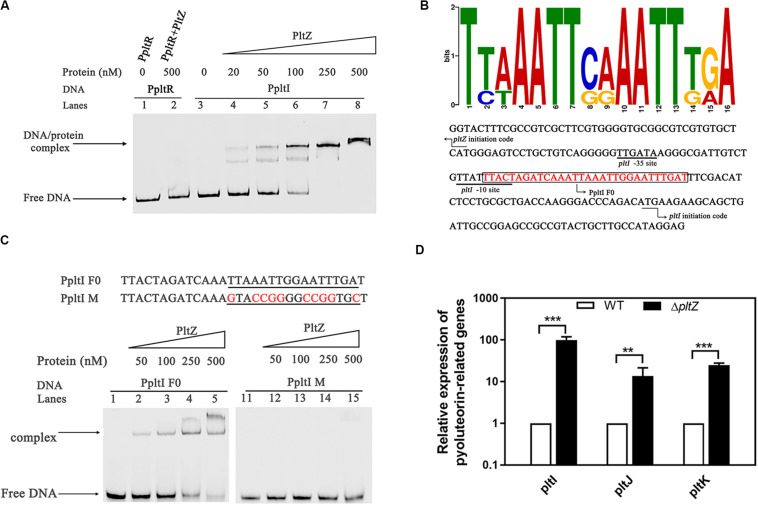
The transcription regulator PltZ binds to the upstream semi-palindromic sequence of the *pltIJKNOP* operon. **(A)** Electrophoretic mobility shift assay (EMSA) of PltZ with PpltI. The probe PpltI (20 ng) was incubated with different amounts of PltZ protein (20–500 nM) in total reaction mixtures of 20 μl each and the probe PpltR (20 ng) was used as the negative control. **(B)** The logo of the PltZ binding motif, which was generated from the conserved 16-nt sequences with the promoter regions of *pltI* and *phlG* from *Pseudomonas aeruginosa* ATCC 27853, *Pseudomonas protegens* Pf-5, and *Pseudomonas fluorescens* F113 using the program MEME (*top*). The *pltI* promoter region of *P. aeruginosa* ATCC 27853 was predicted by the BPROM program (*bottom*). *Black lines* denote the –35 and –10 promoter regions of *pltI*. The PltZ binding motif is shown in *red*. The DNA probe containing the PltZ binding motif (PpltI F0) used for the subsequent EMSA is *boxed*. *Black arrows* indicate the translation start sites of *pltI* and *pltZ*, respectively. **(C)** EMSA of PltZ with the probes PpltI F0 and PpltI M. A total of 20 ng DNA probes was incubated with increasing amounts of the PltZ protein (50–500 nM). PltZ was found to bind to PpltI F0, but not to PpltI M. **(D)** Quantitative real-time reverse transcription PCR (qRT-PCR) assays on *pltI*, *pltJ*, and *pltK* genes showing differences in expression between the wild-type and Δ*pltZ* strains. *Error bars* denote standard deviations (*n* = 3). **p < 0.05, ***p < 0.01.

Since the proteomic data indicated that PltZ negatively regulates the *pltIJKNOP* operon, we were wondering whether this regulation effect is through the direct physical interaction between PltZ and the promoter of the *pltI* gene. DNA fragments comprising the *pltZ*–*pltI* (designated as PpltI) intergenic region and the upstream sequence of *pltR* (designated as PpltR) were amplified by PCR and electrophoretic mobility shift assay (EMSA) was carried out to probe the PltZ–DNA interaction. It was shown that the PltZ bound to PpltI in a concentration-dependent manner, with a saturation concentration of PltZ as low as 250 nM, while no interaction was observed between PltZ and PpltR (Figure 3A). As PltZ shares high sequence identity with PhlH, we reasoned that these two proteins may recognize similar DNA motifs. Using the motif finding program MEME (Bailey and Elkan, 1994), we found that a 16-bp semi-palindromic DNA motif TNNAATTNNAATTNNA is shared among the upstream sequences of PltI and PhlG from *P. aeruginosa* ATCC 27853, *P. protegens* Pf-5, and *Pseudomonas fluorescens* F113 (Figure 3B). This PltZ binding motif was quite similar to the PhlH binding motif previously identified by Yan et al. (2017) and further confirmed by EMSA (Figure 3C). Mutating 10 out of the 16 sites in the predicted PltZ binding motif abolished the PltZ–DNA interaction, confirming the crucial role of the 16-bp semi-palindromic DNA motif in binding PltZ (Figure 3C). By using the BPROM program (Solovyey and Salamov, 2010), a -35/-10 motif (TTGATA/TTATTTACT) recognized by σ^70^ factors was predicted to be present in the upstream of *pltI* (Figure 3B). It is interesting to note that the −10 motif overlaps with the PltZ binding site, suggesting that PltZ represses the *pltIJKNOP* expression by competing with the σ^70^ factors. We further validated the *pltZ*-mediated regulation of *pltIJKNOP* expression by using quantitative real-time reverse transcription PCR (qRT-PCR), and it was revealed that deletion of *pltZ* led to a significant upregulation of the *pltIJK* transcription levels in *P. aeruginosa* ATCC 27853 (Figure 3D). Collectively, these results indicated that PltZ directly represses the expression of the *pltIJKNOP* operon through binding to the conserved semi-palindromic sequence upstream of *pltI*.

### The Antimicrobial 2,4-DAPG Releases the *pltZ*-Mediated Repression of *pltIJKNOP* Expression

**FIGURE 4:**
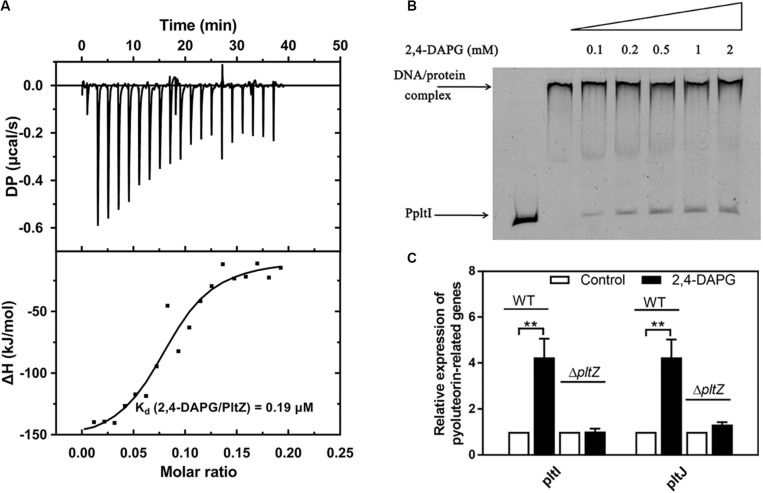
The antimicrobial 2,4-diacetylphloroglucinol (2,4-DAPG) releases the *pltZ*-mediated repression of *pltIJKNOP* expression. **(A)** Interaction between 2,4-DAPG and PltZ was evaluated by isothermal titration calorimetry (ITC). Data were analyzed using the MicroCal PEAQ-ITC software. **(B)** Different concentrations of 2,4-DAPG were added to explore the stability of the PltZ–PpltI complex using electrophoretic mobility shift assay (EMSA). The concentrations of PpltI and PltZ were 20 ng and 0.5 μM, respectively. From *lanes* 3–7, the ligand 2,4-DAPG was added at 0.1, 0.2, 0.5, 1, and 2 mM, respectively. **(C)** Quantitative real-time reverse transcription PCR (qRT-PCR) assays on *pltI* and *pltJ* genes of the wild-type and Δ*pltZ* strains after adding 100 μM 2,4-DAPG to the medium. *P. aeruginosa* cells were grown to logarithmic phase (8 h) for qRT-PCR assays. *Error bars* denote standard deviation (*n* = 3). **p < 0.05.

The TetR family regulators typically recognize small-molecule ligands that modulate their DNA binding capacity. Since PltZ shares ∼39% sequence identity with another TetR family regulator, PhlH, which was shown to bind the antimicrobial 2,4-DAPG (Yan et al., 2017), we speculated that PltZ was likely to also interact with 2,4-DAPG. Using the isothermal titration calorimetry (ITC) assay, we confirmed that the purified PltZ protein could bind 2,4-DAPG with a binding constant of ∼0.19 μM (Figure 4A), which was in the range of the physiological concentration (Yan et al., 2017). Using the EMSA, we further showed that the PltZ/PpltI complex could be partially dissociated by 100 μM 2,4-DAPG (Figure 4B). Moreover, the transcription levels of *pltI* and *pltJ* were markedly increased in the presence of 100 μM 2,4-DAPG, confirming the induction effect of 2,4-DAPG in the *pltIJKNOP* expression (Figure 4C). However, in the Δ*pltZ* strain, there were no significant differences in the mRNA levels of *pltI* and *pltJ* after adding 2,4-DAPG (100 μM) to the medium (Figure 4C). These results confirmed that the induction effect of 2,4-DAPG on the *pltIJKNOP* expression is dependent on the interaction between PltZ and the antimicrobial 2,4-DAPG.

### The PltIJKNOP Efflux Pump Is Involved in the Resistance to Chloramphenicol and Ampicillin in *P. aeruginosa* ATCC 27853

As the proteins PltI, PltJ, and PltP are similar to the three components of the type I secretion system, which is a two-membrane-spanning secretion apparatus similar to the RND efflux pumps commonly related to MDR (Dinesh and Ayush, 2013), we hypothesized that the *pltIJKNOP* operon may be involved in the antibiotic resistance of *P. aeruginosa* ATCC 27853. The wild-type strain showed resistance to a number of clinically relevant antibiotics, including chloramphenicol, ampicillin, tetracycline, kanamycin, and gentamicin. Deletion of *pltI* led to an increased susceptibility to chloramphenicol and ampicillin (Table 1), indicating that the *pltIJKNOP* operon contributes to the intrinsic antibiotic resistance of *P. aeruginosa* ATCC 27853. In contrast, the Δ*pltZ* strain showed a marked increase in the resistance to chloramphenicol and ampicillin (Table 1), consistent with its role in repressing *pltIJKNOP* expression. Interestingly, deletion of *pltI* had no effect on the susceptibility of *P. aeruginosa* ATCC 27853 to 2,4-DAPG (Table 1), suggesting that 2,4-DAPG was not the substrates of the PltIJKNOP efflux pump. Taken together, our data indicated that PltIJKNOP is a drug efflux pump contributing to the antibiotic resistance of *P. aeruginosa*, but the selectivity and action mechanism of this efflux pump await further investigation.

**TABLE 1:** Minimum inhibitory concentrations (MICs) of *Pseudomonas aeruginosa* ATCC 27853 and its mutant strains.

Strains	MICs (μg/ml)					
	Cm	Amp	Ka	Gm	Tet	2,4-DAPG
Δ*pltI*	16	64	1,024	2	16	32
wt	64	256	1,024	4	16	32
Δ*pltZ*	128	1,024	1,024	2	8	–

**wt*, wild-type; *Cm*, chloramphenicol; *Amp*, ampicillin; *Ka*, kanamycin; *Gm*, gentamicin; *Tet*, tetracycline; *2,4-DAPG*, 2,4-diacetylphloroglucinol.*

## Discussion

Antibiotics have been proven to be the most successful therapy to treat infection of microbial pathogens. Thus, the ecological function of antibiotics in nature has long been thought to be helping antibiotic producers outcompete the other microbes through bactericidal or bacteriostatic effects, which is a good example of the Darwinian struggle-for-life theory (Linares et al., 2006). However, in recent years, evidence began to accumulate that certain antibiotics with sublethal or sub-inhibitory concentrations can act as important signaling molecules facilitating intraspecies or interspecies interactions within microbial communities (Romero et al., 2011). One elegantly demonstrated example is that of the sub-inhibitory concentrations of the aminoglycoside tobramycin enhancing the biofilm formation of *P. aeruginosa via* the aminoglycoside response regulator (*arr*) (Hoffman et al., 2005). Here, we found that the antimicrobial 2,4-DAPG, which is produced by the rhizobacterium *P. protegens*, could be recognized by the TetR family regulator PltZ and induces the expression of the *pltIJKNOP* operon encoding a putative efflux pump. It was further shown that the *pltIJKNOP* operon is responsible for the resistance to chloramphenicol and ampicillin. As our proteomic data also revealed that PltZ influences multiple physiological processes of *P. aeruginosa* ATCC 27853, especially the transporting systems of amino acids, glucose, metal ions, and bacteriocins, it is probable that sub-inhibitory concentrations of 2,4-DAPG could serve as a signaling molecule modulating bacterial physiology through allosteric regulation of PltZ.

Pyoluteorin is an antibiotic produced by certain *Pseudomonas* species and suppresses plant diseases caused by the plant pathogen *Pythium ultimum* by inhibiting its growth (Howell, 1980). In *P. protegens* Pf-5, the biosynthesis of pyoluteorin was previously identified to be related to a 24-kb gene cluster named *plt*, within which 10 genes (*pltMRLABCDEFG*) are strictly required for pyoluteorin biosynthesis. Downstream of the *pltG* gene is the *pltZ* gene encoding a TetR family regulator, which was shown to negatively regulate the biosynthetic *pltLABCDEFG* operon (Huang et al., 2004). However, in *P. aeruginosa* ATCC 27853, there are premature stop codons within both *pltR* and *pltB*, making this strain incapable of producing pyoluteorin, which was further confirmed by HPLC. The inactivation of the *pltR* and *pltB* genes implies that the selective constraints imposed upon these two genes are relaxed in *P. aeruginosa* ATCC 27853, in which production of pyoluteorin may provide a few fitness advantages. This seems to be consistent with the clinical background of *P. aeruginosa* ATCC 27853, which was initially isolated from a blood specimen in Peter Bent Brigham Hospital in 1971 (Boston, United States) (Medeiros et al., 1971). Since pyoluteorin typically promotes the fitness of the producers in the rhizosphere where microbial competition is fierce, it is likely that, in clinical settings, pyoluteorin is not beneficial for the survival of *P. aeruginosa* ATCC27853 in human hosts but rather poses a metabolic burden on the producer cells, just as it did in *P. protegens* Pf-5 in laboratory cultivation (Yan et al., 2018).

Despite the biosynthetic pathway of pyoluteorin being disrupted in the ATCC 27853 strain, the *pltIJKNOP* operon, which is located downstream of the *pltLABCDEFG* operon, is fully functional. Sequence analysis indicated that PltI, PltJ, and PltP are highly similar to the three components of the type I secretion system, which spans the inner and outer membranes of Gram-negative microbes and translocates unfolded substrate proteins to the extracellular space in one step. Although it was unknown whether *pltIJKNOP* is involved in secreting certain substrate proteins, we have shown that the *pltI* gene contributes to resistance to several clinically relevant antibiotics. Deletion of the regulatory gene *pltZ* led to a significant increase in the antibiotic resistance of the ATCC 27853 strain, further supporting the role of *pltZIJKNOP* in antibiotic resistance. Since the highly virulent *P. aeruginosa* LESB58 strain also possesses *pltZIJKNOP*, which was found to be incorporated into its genome through horizontal gene transfer (Winstanley et al., 2009), it is possible that *pltZIJKNOP* could be a driving force for the acquired antibiotic resistance in certain clinical strains of *P. aeruginosa*. However, further work needs to be done to establish the substrate spectrum and action mechanism of the PltIJKNOP efflux pump in more detail.

## Materials and Methods

### Bacterial Strains and Culture Conditions

*Escherichia coli* BL21 (DE3) cells were used to express recombinant proteins and *E. coli* DH5α cells were used for molecular cloning. The *E. coli* S17-1 strain was used for conjugation. *E. coli* was grown at 37°C at 220 rpm in Luria–Bertani (LB) broth or agar plates, and *P. aeruginosa* and its derivatives were grown at 37°C at 220 rpm in LB or King’s B (KB) medium (Wang et al., 2019). When required, the growth medium was supplemented with ampicillin (50 mg/L), kanamycin sulfate (50 mg/L), gentamicin (30 mg/L), sucrose (10%, *m*/*v*), and 5-bromo-4-chloro-3-indolyl-β-D-galactopyranoside (X-Gal, 40 mg/L). Strains and plasmids are listed in Supplementary Table S2. The primers and DNA sequences for PCR amplification are listed in Supplementary Table S3.

### Protein Expression and Purification

The protein expression and purification of PltZ were performed as previously described (Wang et al., 2019). Briefly, the *pltZ* gene from *P. aeruginosa* ATCC 27853 was cloned into the pET-28b-derived vector and transformed into the *E. coli* BL21 (DE3) strain. When the optical density at 600 nm of the culture reached ∼0.8, 0.1 mM isopropyl-β-D-thiogalactopyranoside (IPTG) was added and then the bacteria were cultured for another 22 h at 16°C before harvesting. The cells were resuspended in buffer containing 10 mM imidazole, 20 mM Tris–HCl (pH 8.0), and 100 mM NaCl and then sonicated. The cell lysate was centrifuged at 12,000 × *g* for 30 min and then the supernatant was loaded onto a Ni-NTA affinity column. The target protein was eluted with buffer containing 20 mM Tris–HCl (pH 7.5), 100 mM NaCl, and 250 mM imidazole. The imidazole in the protein sample was then removed through multiple rounds of ultrafiltration. The protein was finally concentrated to ∼20 mg/ml and stored at −80°C for further usage.

### Isothermal Titration Calorimetry Assay

The interaction between 2,4-DAPG and PltZ was measured using ITC at 25°C with a MicroCal PEAQ-ITC instrument (Malvern, United States). One millimolar 2,4-DAPG solution used for titration was prepared with the ITC buffer (20 mM Tris, pH 8.0, 100 mM NaCl). Protein samples were prepared by dialysis in the ITC buffer. The concentrations of PltZ and 2,4-DAPG were both 25 μM. The protein and ligand solutions were centrifuged at 12,000 × *g* for 30 min before titration. The titration was performed with a total of 19 injections of 40 μl 2,4-DAPG into the PltZ solution (200 μl). 2,4-DAPG was also titrated into the ITC buffer alone, and the resulting heat of dilution was subtracted from the experimental curve. Data analyses were performed with the MicroCal PEAQ-ITC Analyze software, and an independent binding model was used to calculate the thermodynamic parameters of interaction between PltZ and 2,4-DAPG.

### Quantification of Pyoluteorin

Quantification of pyoluteorin was performed according to the method described previously (Yan et al., 2017). Briefly, HPLC was used to detect the pyoluteorin production of *P. aeruginosa* ATCC 27853. The culture samples were centrifuged at 12,000 × *g* for 10 min and the supernatant was subjected to HPLC analysis. The mobile phases were acetonitrile and distilled water containing 0.1% formic acid. Five percent acetonitrile was used to calibrate the Agilent ZORBAX Eclipse XDB-C18 column, 4.6 × 150 mm. The gradient was performed from 0 to 40% acetonitrile in 3 min and then from 40 to 100% acetonitrile in 3 min, at a flow rate of 1.5 ml/min. The retention time for pyoluteorin was 5.5 min.

### Construction of In-Frame Deletion Mutants of *P. aeruginosa* ATCC 27853

The in-frame deletion mutants were constructed using a two-step homologous recombination method described previously (Wang et al., 2019). Briefly, DNA fragments approximately 1 kb of the upstream and 800 bp of the downstream regions of the target genes were amplified by PCR from *P. aeruginosa* ATCC 27853 genomic DNA using the primers described in Supplementary Table S3. The two fragments were digested with the corresponding restriction enzymes and cloned into the suicide vector pK18mobsacB-Gm. The resulting plasmids were conjugated from *E. coli* S17-1 into *P. aeruginosa* ATCC 27853 by biparental mating. The deletion stains were obtained by selection on LB plates containing Amp, Gm, and 10% sucrose and further confirmed by PCR amplification.

### Total Protein Extraction, Digestion, and MS Analysis

*Pseudomonas aeruginosa* ATCC 27853 wild-type and Δ*pltZ* strains were collected, lysed, reduced, and alkylated at OD_600_ = 1 as previously described (Wang et al., 2019). Briefly, the collected cells were resuspended in 100 μl of cell lysate (8 M urea, 10 mM TCEP, 40 mM CAA, and 10 mM Tris, pH 8.5), followed by boiling in a 95°C water bath for 5 min. The samples were diluted to 1 ml with the dilution buffer (10% ACN and 25 mM Tris, pH 8.5) and the proteins were digested with 10 μg/ml trypsin for 18 h at 37°C and further purified by C18 Stage Tips. Proteomics MS was analyzed by an Orbitrap Fusion Lumos mass spectrometer (Thermo Fisher Scientific, Untied States) coupled online to an EASY-nLC 1200 system. Of the peptide sample, 4 μl was injected into a 15-cm-long, 75-μm inner diameter capillary analytic column packed with C18 particles of 2 μm diameter. The mobile phases for the LC include buffer A (0.1% FA) and buffer B (80% acetonitrile and 0.1% FA). The peptides were separated using a 90-min non-linear gradient consisting of 5–35% buffer B for 60 min, 35–80% buffer B for 20 min, and 100% buffer B for 10 min at a flow rate of 300 nl/min. The source voltage and current were set at 2.5 kV and 100 A, respectively. All mass spectrometry (MS) measurements were performed in the positive ion mode and acquired across the mass range of 300–1,800 *m*/*z*. The raw MS files were analyzed by Proteomics Discovery 2.2 software (Thermo Fisher Scientific, United States) and the MS/MS spectra were searched against the protein sequences of *P. aeruginosa* ATCC 27853. The methionine oxidation and cysteine carbamidomethylation were included as the variable modification and fixed modification, respectively. Other parameters were set up using the default values, and the false discovery rate was set to 0.01 for both peptide and protein identifications. Statistical analysis was done using the Perseus program (Tyanova et al., 2016). Student’s *t*-test was used to determine the significance of the differential expressions of proteins between the Δ*pltZ* and wild-type samples. Proteins within a statistical region of *p* < 0.05 and fold change >2 were considered as differentially expressed proteins. Biological pathway enrichment analysis was performed with the R package clusterProfiler (Yu et al., 2012) using the default settings, showing the 10 most descriptive categories.

### Electrophoretic Mobility Shift Assay

The DNA fragments used for the EMSA were either amplified using PCR or synthesized (Supplementary Table S3). DNA probes were added at 40 ng and incubated at 4°C for 30 min with various amounts of proteins in a total volume of 20 μl mixtures. The mixture contained 50 mM Tris–HCl (pH 7.5), 10 mM MgCl_2_, 10% (*v*/*v*) glycerol, 0.5 mM EDTA, and 50 mM KCl. The samples were subjected to 5% native PAGE with 1 × Tris borate–EDTA (TBE) buffer. Electrophoresis was performed at 120 V, 4°C in an ice-cold bath. The images were acquired by a PharosFX Gel imaging system (Bio-Rad, United States).

### RNA Extraction and Quantitative Real-Time PCR Assay

The qRT-PCR assays were performed as described previously (Yan et al., 2017). Briefly, total RNA of the wild-type and Δ*pltZ* mutants was isolated with Trizol and chloroform. First-strand cDNAs were synthesized using a PrimeScript^TM^ RT reagent kit with gDNA Eraser (TaKaRa, Dalian, China). For real-time PCR analysis, gene-specific primers were used, according to the manufacturer’s instructions (SYBR Premix Ex Taq II kit, TaKaRa, Dalian, China), using 100 ng of total RNA as the template (1.0 μl of cDNA samples). The qRT-PCR assays were performed on a CFX real-time PCR system (Bio-Rad, Hercules, CA, United States). The fold changes of the corresponding gene transcripts in the wild-type strain relative to the Δ*pltZ* strain were computed using the 2^–ΔΔCT^ formula (Livak and Schmittgen, 2001). The 16S rRNA gene was used to normalize the data and at last three biological replicates were performed. Student’s *t*-test was used to compare the gene expression levels, and a *p*-value of 0.05 was considered statistically significant.

### MIC Determination

Minimum inhibitory concentration (MIC) analysis was carried out in 96-well microtiter plates with the standard broth microdilution method (Schwalbe et al., 2007). The bacteria used in the study were those harvested in the mid-exponential growth phase (absorbance at 600 nm was 0.6) and diluted 1,000-fold with the LB broth. Of the diluted bacterial culture, 100 μl was added to each well; the MIC values were determined as the lowest concentration that completely inhibited bacterial growth after 20 h of incubation at 37°C.

## Data Availability Statement

The datasets generated for this study can be found in the ProteomeXchange Consortium (Vizcaíno et al., 2016) with the data set identifier PXD016202 (http://www.proteomexchange.org).

## Author Contributions

Y-XH conceived and designed the experiments. L-ML performed the experiments. H-LM, S-PZ, HX, HZ, YW, YY, and ZW analyzed the data. Y-XH and D-DG wrote the manuscript. All authors contributed to the article and approved the submitted version.

## Conflict of Interest

The authors declare that the research was conducted in the absence of any commercial or financial relationships that could be construed as a potential conflict of interest.
