# Supplementary material for: The Regulator PltZ Regulates a Putative ABC Transporter System PltIJKNOP of Pseudomonas aeruginosa ATCC 27853 in Response to the Antimicrobial 2,4-Diacetylphloroglucinol
Source: Front Microbiol. 2020 Jul 8;11:1423. doi: 10.3389/fmicb.2020.01423 (PMC7360687; doi:10.3389/fmicb.2020.01423)
Supplement: Supplementary file 1 [file Data_Sheet_1.docx]

**Supplementary Table S1 up- and down- regulated proteins in Δ*pltZ* samples**

| P-value | log_2_(Fold Change) | Description | |
| --- | --- | --- | --- |
| **Up-regulated** |  |  |  |
| 0.0312 | 2.30674 | Ga0133450_111007 | PA3924 fatty-acyl-CoA synthase |
| 0.0457 | 1.07267 | Ga0133450_111012 | PA3919 PhoH-like ATPase |
| 0.0259 | 2.76812 | Ga0133450_111069 | dauA D-arginine dehydrogenase |
| 0.0035 | 3.31566 | Ga0133450_111070 | dauB ornithine cyclodeaminase |
| 0.038 | 2.06989 | Ga0133450_111134 | PA3801 Putative negative regulator of RcsB-dependent stress response |
| 0.0281 | 2.54811 | Ga0133450_111177 | mltF membrane-bound lytic murein transglycosylase F |
| 0.0235 | 3.16109 | Ga0133450_111198 | rimM 16S rRNA processing protein RimM |
| 0.0064 | 2.46278 | Ga0133450_111218 | PA3726 Uncharacterized conserved protein YaeQ, suppresses RfaH defect |
| 0.0478 | 1.05621 | Ga0133450_111244 | PA3699 transcriptional regulator, TetR family |
| 0.0043 | 4.71535 | Ga0133450_111271 | PA3672 ABC-2 type transport system ATP-binding |
|  |  |  | protein |
| 0.0325 | 3.02207 | Ga0133450_111273 | PA3670 ABC-type uncharacterized transport system involved in gliding motility, auxiliary component |
| 0.0402 | 1.81744 | Ga0133450_1113 | glyQ glycyl-tRNA synthetase alpha chain |
| 0.0275 | 3.84916 | Ga0133450_111308 | phage replication protein P |
| 0.0313 | 2.22577 | Ga0133450_11134 | PA0126 hypothetical protein |
| 0.008 | 2.06832 | Ga0133450_111365 | PA3641 alanine or glycine: cation symporter, AGCS |
|  |  |  | family |
| 0.0463 | 3.06917 | Ga0133450_11169 | opdC outer membrane porin, OprD family |
| 0.031 | 1.22829 | Ga0133450_111699 | PA3301 Lysophospholipase, alpha-beta hydrolase |
|  |  |  | superfamily |
| 0.0158 | 2.76966 | Ga0133450_111724 | PA3277 NADP-dependent 3-hydroxy acid |
|  |  |  | dehydrogenase YdfG |
| 0.0062 | 4.09414 | Ga0133450_111731 | PA3271 Na+/proline symporter |
| 0.0179 | 2.21735 | Ga0133450_111747 | hypothetical protein |
| 0.0427 | 2.94795 | Ga0133450_11177 | PA0170 protein of unknown function (DUF1987) |
| 0.0016 | 3.33998 | Ga0133450_111777 | PA3230 hypothetical protein |
| 0.0443 | 1.13555 | Ga0133450_111782 | PA3225 DNA-binding transcriptional regulator, LysR |
|  |  |  | family |
| 0.0289 | 1.21938 | Ga0133450_111793 | PA3214 cholesterol transport system auxiliary |
|  |  |  | component |
| 0.0137 | 3.65169 | Ga0133450_111815 | glk glucokinase |
| 0.0007 | 4.11793 | Ga0133450_111819 | PA3189 glucose ABC transporter membrane protein |
| 0.0088 | 1.01409 | Ga0133450_111821 | PA3187 glucose ABC transporter ATP-binding protein |
| 0.0403 | 1.80669 | Ga0133450_111827 | eda 2-keto-3-deoxy-phosphogluconate aldolase |
| 0.0029 | 2.45217 | Ga0133450_111846 | cmk cytidylate kinase |
| 0.0192 | 1.17784 | Ga0133450_111864 | uvrB Excinuclease ABC subunit B |
| 0.0002 | 3.59875 | Ga0133450_111874 | PA3130 acyl-CoA thioester hydrolase |
| 0.0448 | 2.12698 | Ga0133450_111902 | xcpR type II secretion system protein E (GspE) |
| 0.0076 | 2.11338 | Ga0133450_111926 | PA3081 Protein of unknown function (DUF1329) |
| 0.0006 | 2.56632 | Ga0133450_111962 | PA3046 hypothetical protein |
| 0.0204 | 3.89025 | Ga0133450_111965 | dgt2 dGTPase |
| 0.0011 | 3.18257 | Ga0133450_111966 | PA3042 hypothetical protein |
| 0.0233 | 2.29635 | Ga0133450_111996 | fada5 3-ketoacyl-CoA thiolase |
| 0.0146 | 2.35867 | Ga0133450_112038 | rluC ribosomal large subunit pseudouridine synthase C |
| 0.0101 | 4.49487 | Ga0133450_112076 | PA2942 protoporphyrin IX magnesium-chelatase |
| 0.0025 | 3.86048 | Ga0133450_1121 | rsmB 16S rRNA m (5) C-967 methyltransferase |
| 0.0469 | 2.69931 | Ga0133450_112101 | PA2918 NAD(P)-dependent dehydrogenase, short-chain alcohol dehydrogenase family |
| 0.0251 | 2.47915 | Ga0133450_112170 | PA2852 conserved hypothetical protein, PP_1857 |
|  |  |  | family |
| 0.0019 | 3.20143 | Ga0133450_112190 | tpm thiopurine S-methyltransferase |
| 0.0096 | 1.15131 | Ga0133450_112199 | PA2823 hypothetical protein |
| 0.0026 | 1.79763 | Ga0133450_112314 | Superfamily I DNA and/or RNA helicase |
| 0.0333 | 4.15623 | Ga0133450_112397 | nuoL NADH dehydrogenase subunit L |
| 0.0418 | 1.16687 | Ga0133450_112430 | lolA outer membrane lipoprotein carrier protein |
| 0.0002 | 3.17386 | Ga0133450_112528 | HlyD family secretion protein |
| 0.0024 | 3.02116 | Ga0133450_112529 | ABC-2 type transport system ATP-binding protein |
| 0.0008 | 3.03451 | Ga0133450_112530 | ABC-2 type transport system permease protein |
| 0.017 | 4.25135 | Ga0133450_112531 | ABC-2 type transport system permease protein |
| 0 | 3.27782 | Ga0133450_112532 | efflux transporter, outer membrane factor (OMF) lipoprotein, NodT family |
| 0.0277 | 1.06684 | Ga0133450_112566 | Protein of unknown function (DUF3141) |
| 0.0078 | 3.38427 | Ga0133450_112617 | DNA binding domain-containing protein, excisionase |
|  |  |  | family |
| 0.0124 | 1.49307 | Ga0133450_112713 | xthA Exodeoxyribonuclease III |
| 0.0008 | 2.15499 | Ga0133450_112798 | PA2463 hemolysin activation/secretion protein |
| 0.014 | 2.24453 | Ga0133450_11282 | PA0268 transcriptional regulator, GntR family |
| 0.0033 | 3.02283 | Ga0133450_11283 | PA0269 alkylhydroperoxidase AhpD family core |
|  |  |  | domain-containing protein |
| 0.0472 | 2.98788 | Ga0133450_113124 | PA2201 protein of unknown function (DUF1911) |
| 0.0236 | 4.141 | Ga0133450_113156 | PA2166 Protein of unknown function (DUF3509) |
| 0.0001 | 3.57818 | Ga0133450_11320 | spuH putrescine transport system permease protein |
| 0.0038 | 3.26005 | Ga0133450_113376 | rbsC monosaccharide ABC transporter membrane protein, CUT2 family |
| 0.0405 | 2.58953 | Ga0133450_113509 | nhaB sodium/proton antiporter, NhaB family |
| 0.0237 | 2.90812 | Ga0133450_113522 | PA1807 microcin C transport system ATP-binding |
|  |  |  | protein |
| 0.0275 | 4.11451 | Ga0133450_113560 | cmpX Conserved TM helix |
| 0.0019 | 3.07826 | Ga0133450_113590 | PA1745 hypothetical protein |
| 0.0073 | 3.05241 | Ga0133450_11360 | PA0339 Uncharacterized conserved protein, contains NRDE domain |
| 0.0225 | 1.66047 | Ga0133450_113647 | PA1689 Phosphoglycerol transferase MdoB |
| 0.0236 | 2.11644 | Ga0133450_113654 | mtnB methylthioribulose-1-phosphate dehydratase |
| 0.0281 | 3.09299 | Ga0133450_113683 | PA1655 glutathione S-transferase |
| 0.0003 | 3.5658 | Ga0133450_113692 | PA1646 methyl-accepting chemotaxis protein |
| 0.0466 | 1.87843 | Ga0133450_113699 | PA1640 NTE family protein |
| 0.0135 | 2.6549 | Ga0133450_113705 | kdpB K+-transporting ATPase ATPase B chain |
| 0 | 3.27024 | Ga0133450_113728 | PA1611 Signal transduction histidine kinase |
| 0.0407 | 3.20508 | Ga0133450_113735 | PA1604 amino acid ABC transporter substrate-binding protein, PAAT family |
| 0.0477 | 2.72546 | Ga0133450_11377 | PA0356 HD-like signal output (HDOD) domain, no enzymatic activity |
| 0.003 | 3.48682 | Ga0133450_113780 | cobalt-zinc-cadmium resistance protein CzcA |
| 0.0223 | 2.28995 | Ga0133450_113821 | PA1566 glutamate--putrescine ligase |
| 0.043 | 2.41122 | Ga0133450_113957 | rnd ribonuclease D |
| 0.004 | 2.91925 | Ga0133450_114003 | ccmC heme exporter protein C |
| 0.0286 | 1.97483 | Ga0133450_114024 | cheY two-component system, chemotaxis family, response regulator CheY |
| 0.0203 | 1.99229 | Ga0133450_11404 | trmB tRNA (guanine-N (7)-)-methyltransferase |
| 0.0312 | 2.22975 | Ga0133450_11426 | pyrR pyrimidine operon attenuation protein / uracil phosphoribosyltransferase |
| 0.0006 | 3.43586 | Ga0133450_114294 | fliG flagellar motor switch protein FliG |
| 0.0236 | 2.75205 | Ga0133450_114302 | flagellar protein FliS |
| 0.0003 | 3.12816 | Ga0133450_114307 | methyltransferase, FkbM family |
| 0.032 | 1.39242 | Ga0133450_114312 | 3-oxoacyl-[acyl-carrier-protein] synthase-3 |
| 0.0027 | 3.74759 | Ga0133450_114316 | flagellar hook-associated protein 1 FlgK |
| 0.0001 | 3.31711 | Ga0133450_114421 | queC preQ (0) biosynthesis protein QueC |
| 0.002 | 4.43755 | Ga0133450_114436 | cold-shock DNA-binding protein family |
| 0.005 | 1.41419 | Ga0133450_114467 | cysM cysteine synthase B |
| 0.0074 | 2.36017 | Ga0133450_114473 | PA0926 hypothetical protein |
| 0.0047 | 4.00047 | Ga0133450_114483 | kup KUP system potassium uptake protein |
| 0.043 | 2.17444 | Ga0133450_114509 | argR transcriptional regulator, AraC family with amidase-like domain |
| 0.0073 | 2.71539 | Ga0133450_114547 | bolA transcriptional regulator, BolA protein family |
| 0.0009 | 3.39933 | Ga0133450_114554 | PA0850 MAPEG family protein |
| 0.0005 | 3.75045 | Ga0133450_114578 | PA0827 hypothetical protein |
| 0.046 | 2.83309 | Ga0133450_114614 | histidinol-phosphatase (PHP family) |
| 0.0012 | 3.18266 | Ga0133450_114639 | PA0804 Aldo/keto reductase |
| 0.0028 | 3.45447 | Ga0133450_114674 | rnc RNAse III |
| 0.0391 | 1.60537 | Ga0133450_114682 | algU RNA polymerase, sigma-24 subunit, RpoE |
| 0.0132 | 1.48714 | Ga0133450_114695 | ung Uracil-DNA glycosylase |
| 0.024 | 1.9044 | Ga0133450_114698 | PA0747 methylmalonate-semialdehyde dehydrogenase |
| 0.0005 | 1.02596 | Ga0133450_114799 | thioredoxin |
| 0.0092 | 3.73006 | Ga0133450_114987 | PA4327 Tetratricopeptide repeat-containing protein |
| 0.0498 | 2.17612 | Ga0133450_115061 | panE ketopantoate reductase |
| 0.0064 | 1.0369 | Ga0133450_115107 | cysNC adenylylsulfate kinase /sulfate adenylyltransferase subunit 1 |
| 0.0186 | 2.61582 | Ga0133450_115114 | hisG ATP phosphoribosyltransferase (homohexameric) |
| 0.0079 | 3.46819 | Ga0133450_115120 | PA4454 phospholipid/cholesterol/gamma-HCH transport system substrate-binding protein |
| 0.0283 | 3.24936 | Ga0133450_115121 | PA4455 phospholipid/cholesterol/gamma-HCH transport system permease protein |
| 0.0219 | 2.45049 | Ga0133450_115140 | PA4473 ribosome-associated protein |
| 0.0117 | 3.77119 | Ga0133450_115172 | PA4505 dipeptide transport system ATP-binding |
|  |  |  | protein |
| 0.0063 | 2.28877 | Ga0133450_115188 | PA4521 AmpE protein |
| 0.007 | 3.1981 | Ga0133450_115211 | chromosome partitioning related protein ParA |
| 0.0294 | 3.00653 | Ga0133450_115223 | hypothetical protein |
| 0.0001 | 3.21059 | Ga0133450_11526 | PA0502 pimeloyl-[acyl-carrier protein] methyl ester |
|  |  |  | esterase |
| 0.0419 | 1.32253 | Ga0133450_115364 | PA4571 thiosulfate dehydrogenase |
| 0.0191 | 2.38635 | Ga0133450_115415 | PA4619 Cytochrome c, mono- and diheme variants |
| 0.0013 | 4.27592 | Ga0133450_115436 | hypothetical protein |
| 0.0115 | 1.53461 | Ga0133450_115477 | N-6 DNA Methylase |
| 0.0087 | 3.18388 | Ga0133450_115533 | PA4702 Pentapeptide repeat-containing protein |
| 0.0221 | 2.16259 | Ga0133450_115595 | carA carbamoyl-phosphate synthase small subunit |
| 0.0182 | 2.11993 | Ga0133450_115702 | retS Signal transduction histidine kinase |
| 0.047 | 3.00614 | Ga0133450_115718 | PA4872 carboxyvinyl-carboxyphosphonate |
|  |  |  | phosphorylmutase |
| 0.002 | 5.03584 | Ga0133450_115794 | amiB N-acetylmuramoyl-L-alanine amidase |
| 0.0367 | 2.67011 | Ga0133450_115819 | PA4972 Protein of unknown function (DUF3298) |
| 0.0109 | 3.01013 | Ga0133450_115836 | waaA 3-deoxy-D-manno-octulosonic-acid transferase |
| 0.0014 | 2.79263 | Ga0133450_115877 | PA5028 chromosome partitioning protein |
| 0.0006 | 4.35167 | Ga0133450_115889 | aroK shikimate kinase |
| 0.0247 | 2.23232 | Ga0133450_115902 | PA5052 Cell division protein FtsN |
| 0.0022 | 3.73869 | Ga0133450_115903 | hslV HslV component of HslUV peptidase. Threonine peptidase. MEROPS family T01B |
| 0.0298 | 2.41016 | Ga0133450_115924 | glnq3 amino acid ABC transporter ATP-binding protein, PAAT family |
| 0.0337 | 2.42557 | Ga0133450_115938 | PA5089 hypothetical protein |
| 0.0089 | 1.83404 | Ga0133450_115943 | PA5094 glycine betaine/proline transport system ATP-binding protein |
| 0.0428 | 3.15349 | Ga0133450_115988 | PA5139 amino acid ABC transporter substrate-binding protein, PAAT family |
| 0.0332 | 5.10857 | Ga0133450_116003 | hypothetical protein |
| 0.0233 | 1.03551 | Ga0133450_116024 | rmlD dTDP-4-dehydrorhamnose reductase |
| 0.0039 | 3.36059 | Ga0133450_11603 | dnaG DNA primase |
| 0.0176 | 3.61755 | Ga0133450_116077 | gcvH2 glycine cleavage system H protein |
| 0.02 | 1.38235 | Ga0133450_116158 | PA5291 choline/glycine/proline betaine transport |
|  |  |  | protein |
| 0.0484 | 3.24039 | Ga0133450_116165 | xpt xanthine phosphoribosyltransferase |
| 0.0198 | 3.27521 | Ga0133450_116205 | spoT GTP pyrophosphokinase |
| 0.0439 | 2.38557 | Ga0133450_116216 | alkT rubredoxin-NAD+ reductase |
| 0.0376 | 2.25045 | Ga0133450_116221 | glcE glycolate oxidase FAD binding subunit |
| 0.0004 | 4.36704 | Ga0133450_116235 | pstC phosphate transport system permease protein |
| 0.0141 | 1.84058 | Ga0133450_116236 | psts3 phosphate ABC transporter substrate-binding protein, PhoT family |
| 0.0069 | 1.233 | Ga0133450_116245 | betA choline dehydrogenase |
| 0.0472 | 3.80885 | Ga0133450_116254 | hypothetical protein |
| 0.007 | 5.21191 | Ga0133450_116271 | stcD 2,4-dienoyl-CoA reductase |
| 0.0061 | 3.98672 | Ga0133450_116296 | PA5422 glucose-6-phosphate 1-epimerase |
| 0.0048 | 4.17331 | Ga0133450_116307 | PA5433 Protein N-acetyltransferase, RimJ/Rim family |
| 0.0356 | 2.49739 | Ga0133450_116311 | PA5437 DNA-binding transcriptional regulator, LysR |
|  |  |  | family |
| 0.0145 | 2.90535 | Ga0133450_116330 | rmd GDP-4-dehydro-6-deoxy-D-mannose reductase |
| 0.0313 | 2.71729 | Ga0133450_116348 | PA5472 amino acid ABC transporter substrate-binding protein, PAAT family |
| 0.0001 | 3.3297 | Ga0133450_116355 | gltP proton glutamate symport protein |
| 0.0156 | 1.25935 | Ga0133450_116358 | algB Two-component response regulator AlgB |
| 0.0418 | 3.28336 | Ga0133450_116399 | PA5522 glutamate--putrescine ligase |
| 0.0474 | 3.07156 | Ga0133450_116400 | PA5523 glutamate-1-semialdehyde 2,1-aminomutase |
| 0.0022 | 1.70516 | Ga0133450_116405 | PA5528 Predicted lipid-binding transport protein, |
|  |  |  | Tim44 family |
| 0.0489 | 1.27674 | Ga0133450_116445 | yidC protein translocase subunit yidC |
| 0.0417 | 2.71614 | Ga0133450_1176 | PA0068 hypothetical protein |
| 0.0004 | 3.66764 | Ga0133450_1182 | ppkA serine/threonine-protein kinase PpkA |
| 0.0006 | 3.41645 | Ga0133450_11919 | PA4011 undecaprenyl-diphosphatase |
| 0.0361 | 3.32534 | Ga0133450_11927 | pbpA peptidoglycan glycosyltransferase /celelongation-specific peptidoglycan D, D-transpeptidase |
| 0.0108 | 1.59083 | Ga0133450_11944 | lnt Apolipoprotein N-acyltransferase |
| **Down** |  |  |  |
| **-regulated** |  |  |  |
| 0.0014 | -2.35308 | Ga0133450_111212 | hypothetical protein |
| 0.0051 | -1.28596 | Ga0133450_112191 | PA2831 Murein tripeptide amidase MpaA |
| 0.0104 | -1.31971 | Ga0133450_113768 | trbJ1 P-type conjugative transfer protein TrbJ |
| 0.0203 | -2.07067 | Ga0133450_114323 | flgD flagellar basal-body rod modification protein |
|  |  |  | FlgD |
| 0.0491 | -2.44993 | Ga0133450_116026 | rmlC dTDP-4-dehydrorhamnose 3,5-epimerase |
| 0.0138 | -1.34744 | Ga0133450_116182 | rpmG LSU ribosomal protein L33P |
| 0.0249 | -1.47789 | Ga0133450_116215 | hupA bacterial nucleoid protein HU alpha subunit |

**Supplementary Table S2. Strains and plasmids.**

| Strains or plasmids | Description | Source |
| --- | --- | --- |
| Strains  *E. coli* DH5α | F^–^ *endA1* *glnV44* *thi-1* *recA1* *relA1* *gyrA96* *deoR* *nupG* *purB20*φ80d*lacZ*ΔM15Δ (*lacZYA-argF*) U169, hsdR17 (*r_K_*^–^*m_K_*^+^), λ^–^ | Novagen |
| S17-1  BL21 (DE3)  *P. aeruginosa*  ATCC 27853 | F^–^ *thi pro hsdR* [RP4-2 Tc::Mu Km::Tn7 (TpSm) ]  F^–^ *ompT gal dcm lon hsdSB* (*rB^–^mB^–^*) λ (DE3 [*lacI*  *lacUV5-T7p07 ind1 sam7 nin5*]) [*malB^+^*] _K-12_ (λS)  Wild type; Ap^r^ | Novagen  Novagen  Novagen |
| Δ*pltZ*  Plasmids  pET-28b  pET28*-pltZ*  pK18mobsacB  *-*Gm  pK18mobsacB  *-pltZ-*Gm | *pltZ* gene in-frame deletion in strain ATCC27853; Ap^r^  Expression vector; Km^r^  Plasmid for expression of PltZ; Km^r^  Suicide vector for generation of gene in-frame deletions, *sacB*, Gm^r^  pK18mobsacB derivative for generation of *pltZ* deletion, *sacB*, Km^r^ | This study  Novagen  This study  This study  This study |

**Supplementary Table S3. Primers used in this study.**

| Primers | DNA targets | Sequences90 ((5’ to 3’) |
| --- | --- | --- |
| Primers for expression  in BL21 (DE3) |  |  |
| PltZ-F  PltZ-R | *pltZ* | CACCATCACCATCACCATATGATGAGCACACGACGCCGCCGCGGCCTTTTTGGCCATATGTCAGGAGGGGTCGTGGTG |
| Primers for mutant construction |  |  |
| ∆pltZ-F1  ∆pltZ-R1  ∆pltZ-F2  ∆pltZ-R2  Primers for EMSA assays  PpltI-F  PpltI-R | *pltZ*  *pltI* | CCGGAATTCAGTTGCCGCTCGTTGACATAG  TGCAGCAGGATCGCTTGCATATAACCGTGCTGGGCGAA  TGTTCGCCCAGCACGGTTATATGCAAGCGATCCTGCTGC  TGCTCTAGATACATGATCGTCGACCGGG  Fam-GGTACTTTCGCCGTCGCTT CTCCTATGGCAAGCAGTACGG |
| PpltI F0-F  PpltI F0-R  PpltI M-F  PpltI M-F  PpltR-F  PpltR-R | PpltI F0  PpltI M  PpltR | Fam-TTACTAGATCAAATTAAATTGGAATTTGAT  ATCAAATTCCAATTTAATTTGATCTAGTAA  Fam-TTACTAGATCAAAGTACCGGGGCCGGTGCT  AGCACCGGCCCCGGTACTTTGATCTAGTAA  Fam-CCCTGAACGACAGGATTGC  TTGTGTTGCAGGCAGGTGT |
| Primers for qRT-PCR  pltI-F  pltI-R  pltJ-F  pltJ-R  pltK-F | *pltI*  *pltJ*  *pltK* | ATGAAGAAGCAGCTGATTGCC  AACACCCGGATCTCATAGACC  CGCCCAGGTCTTCGTGTTGTA  ACGATATGCTCCCGGTCA  GCTGTTCCGCGTACCGAT |
| pltK-R |  | GCGGATGAAGGTCGGCACGTT |
